# Detection of Local Tissue Reactions after Anti-GnRF Injection in Male Pigs Assessed Using Magnetic Resonance Imaging

**DOI:** 10.3390/ani11040968

**Published:** 2021-03-31

**Authors:** Maren Bernau, Sebastian Schwanitz, Lena Sophie Kreuzer, Armin Manfred Scholz

**Affiliations:** 1 Livestock Center Oberschleissheim, Veterinary Faculty of the Ludwig-Maximilians-University Munich, St. Hubertusstrasse 12, 85764 Oberschleissheim, Germany; sebastian.schwanitz@lmu.de (S.S.); lena.kreuzer@lmu.de (L.S.K.); armin.scholz@lvg.vetmed.uni-muenchen.de (A.M.S.); 2 Hochschule für Wirtschaft und Umwelt Nürtingen-Geislingen, Fakultät Agrarwirtschaft, Volkswirtschaft und Management, Neckarsteige 6-10, 72622 Nürtingen, Germany

**Keywords:** local reaction, anti-GnRF, pigs, magnetic resonance imaging

## Abstract

**Simple Summary:**

Local tissue reactions after injection are known in animals and humans. This study analysed the local tissue reaction after injection of the so-called immunologic castration in pigs using magnetic resonance imaging to achieve insight into the reaction site. Long-lasting reactions, which can be traced to inflammatory reactions, were detected. Further studies are needed to evaluate the impact of these reactions on animal well-being and carcass quality.

**Abstract:**

This study aimed at evaluating the local tissue reaction of an anti-GnRF product, which is used for the so called “immunocastration” in male pigs. A total of 34 pigs were injected two times (including a booster injection) with an anti-GnRF product. Injection was performed using the prescribed safety vaccinator. Injection sites were evaluated three times post injection using magnetic resonance imaging. Two examinations were performed after the first injection and one after the booster. The local tissue reaction was evaluated three-dimensionally by semi-automatic analyses, by linear measurements of injection depth and length, and by scoring the kind of tissue affected. Results showed a long-lasting reaction in affected muscle tissue. Therefore, needle length should be discussed, and an evaluation of the injection site after slaughter should be performed include behavioural scorings post injection to evaluate the impact on animal well-being and carcass quality.

## 1. Introduction

Immunological castration is considered as a possible solution for piglet castration [1,2,3,4] and is used in other farm animals [5,6,7,8], even in females [9,10,11]. Advantages seem to predominate: no surgical castration has to be performed and housing and behavioural problems which occur during the fattening of male pigs seems to be solved [12,13,14]. Immunologic castration simply involves two injections, but from reading the summary of the product characteristics, side effects were reported [15]: Administration with 8 weeks of age lead to a swelling of up to 4 × 8 cm at the injection site, which may persist for more than 42 days in 20–30% of the animals. Administration with 14 to 23 weeks of age leads to swellings of 2 to 5 cm in diameter.

These large local reactions might affect the animals’ well-being and may affect carcass quality. In order to detect local reactions after administration, an objective method for allowing repeated evaluations post injection in vivo could help evaluate animal welfare issues. In human medicine, magnetic resonance imaging (MRI) is used for the diagnosis and follow-up of musculoskeletal diseases [16,17] and is the method of choice for soft-tissue imaging [18,19,20]. MRI allows the detection of various conditions like trauma, infection or inflammation. In animals, MRI can be used to detect antigen clearance or in pharmacological research [21,22,23,24,25,26,27]. 

The aim of this study was to evaluate the local tissue reaction after injection of an anti-GnRF product to assess the tissue tolerability of this alternative method of piglet castration. MRI was used repeatedly post injection to characterize the local tissue reaction in three-dimensions and its progression over time.

## 2. Materials and Methods

### 2.1. Animal Keeping and Handling

The experimental set-up, including housing and feeding, was conducted in accordance with the District Government of Upper Bavaria (registration number: 55.2-1-54-2532.2-12-13) and local and national guidelines [28,29]. All pigs were hybrids of Piétrain sires mated with German Landrace sows. Piglets were born and raised under conventional pig farming conditions and were kept in groups. To satisfy their natural behavior, all boxes contained balls, metal chains, and diverse chewable material for enrichment. The experimental part was performed with 34 animals divided into three experimental groups (I: *n* = 12; II: *n* = 11; III: *n* = 11).

**Figure 1:**
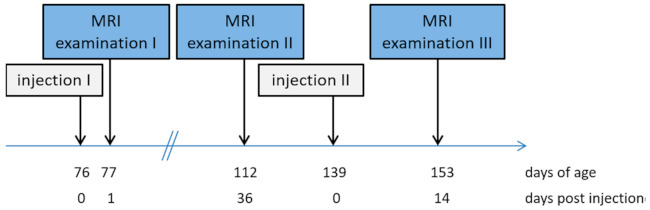
Sequence of the examination procedures.

The pigs (*n* = 34) received two injections of the anti-GnRF product: the first injection at day 76 ± 1 of age and the second (booster) at 139 ± 1 days of age (see Figure 1). Both injections (2 mL) were performed into the right side of the neck using the prescribed safety vaccinator (needle length 19 mm). From birth, all necessary injections were given into the ham muscle only to avoid local reactions in the neck muscle of the pigs. 

### 2.2. Magnetic Resonance Imaging

Magnetic resonance imaging using an open low-field MRI system (Siemens Magnetom Open; field strength 0.2 Tesla) was performed three times during this study. In order to guaranty excellent image quality, the pigs were anaesthetized during the MRI scans. Anaesthesia was performed using a combination of Azaperone (2 mg/kg body weight) and Ketamine (10–15 mg/kg body weight) given intramuscularly into the ham muscle (Musculus semimembranosus, Musculus semitendinosus). All pigs were bedded in a prone position with front limbs flexed and hind limbs extended. Prone bedding prohibits bedding-related abnormalities in dorsal neck MR images. The MRI scans were performed three times post injection, at day 1 (scan 1; average weight 27.1 ± 5.1 kg) and day 36 (scan 2; average weight 51.7 ± 8.7 kg) after the first injection and at day 14 (scan 3; average weight 91.6 ± 11.1 kg) after the second injection. Due to technical disturbances at scan 3 only 22 animals were examined. A T1-weighted spin–echo-sequence with coronar positioning was used. The sequence parameters are shown in Table 1. Due to the repetitive MRI measurements and the fast growth of the pigs, two MRI sequence protocols adapted to the animals’ sizes had to be used for MR imaging. According to body size, the small (scan 1) and the large body coil (scans 2–3) were used as a coil receiver.

### 2.3. Image Analysis

For image evaluation, the Able 3D Doctor^®^ Software (Able Inc., Lexington, MA, USA) was used. Three evaluations were performed.

**Figure 2:**
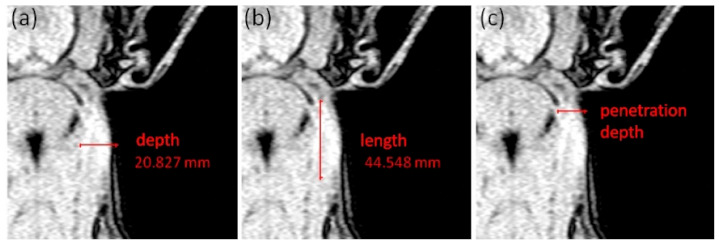
Description of the linear measurements at scan 1, showing a mild local reaction at the right neck side: (**a**) measurement of the maximum depth (mm) of local reaction site, (**b**) measurement of the maximum length (mm) of local reaction and (**c**) measurement of the penetration depth (mm) at the beginning of the signal increased region behind the ear.

1. Volume measurement: Tissues with increased signal intensity were defined as having a local-tissue reaction. A rectangle Region of Interest (ROI) was defined by encircling the largest extent of local reaction at the injection side (IS; see [24]). Inside this ROI, the area with increased signal intensity was bordered using the Interactive Segmentation Function, which allows the segmentation of regions by separating them according to defined grayscales. After bordering the area with increased signal intensity at the IS, the ROI was mirrored to the control side (CS) and the Interactive Segmentation Function applying the same grayscales was used again. By evaluating 5 slices showing increased signal intensities, a volume of the IS and of the corresponding CS was created for images of scan 1 to scan 3. The differences was formed from both volumes (Vol_diff (cm^3^)).
2. Length and depth measurements: Additionally, the MR image with the largest area of hyperintense tissue of each animal and each examination day was analyzed regarding the maximum extent of local reaction in depth and length (in mm; see Figure 2a,b). Therefore, the deepest point at the IS showing a bright signal increase was measured from the skin side and represents the maximum depth (mm) of the local reaction (Figure 2a). The maximum length (mm) of the local reaction was calculated by measuring the cranio-caudal distribution of the bright signal increased region (Figure 2b). Furthermore, the injection site was defined as the point, where the signal increase starts near the ear base. At that point, the penetration depth was measured (in mm; see Figure 2c).
3. Location scoring: Besides the volume and linear measurements, a scoring system was used to describe the location of the local reaction inside the MR images: 0 = subcutaneous; 1 = superficial intramuscular; and 2 = intramuscular.

### 2.4. Statistical Analysis

For the statistic evaluation, different procedures using SAS (version 9.3, SAS Inst., Inc., Cary, NC, USA) were used. To evaluate the volume of the local reaction, an F-Test and a paired *t*-Test were performed to identify significant differences between the volume of the signal increased region at the IS and its corresponding volume at the CS (Table 2). The significance level was set at *p* < 0.05. For the linear measurements and the location scoring the mean values and the standard deviations were calculated (Table 3).

## 3. Results

The results for the volume difference (Vol_diff) between the IS and CS are shown in Table 2. Significant differences between the IS and CS were detected on all examination days. The maximum Vol_diff was achieved at Scan 2 (3.36 ± 0.72 cm^3^, 36 days post injection).

As demonstrated in Table 3, in nearly all cases the injection penetrated the muscular tissue and resulted in a local reaction in the muscular tissue (location score > 1.7). The maximum mean length and depth varied among the scans at a low level. Scan 3 showed the largest extent of local reaction in depth and length, representing day 14 after the booster injection. The local reaction increased from scan 1 to scan 2, which could be detected for the Vol_diff measurements as well. The penetration depth was measured in 14.3 ± 3.8 mm under the skin surface.

## 4. Discussion

Injection of the immunologic product results in local tissue reactions detectable by MRI, which is confirmed in previous studies dealing with MRI as an alternative method for safety testing [25,27]. MRI offers a three-dimensional repetitive monitoring of local reaction sizes in vivo in animals of different age and weight groups [24,25,26,27]. Alterations with an increased signal intensity in T1-weighted MR images can represent inflammatory tissue, fatty infiltration or hematoma [30,31]. Signal intensity changes post injection can be traced back to inflammatory reactions [25,27] and especially in later progress to fibrotic changes like fatty infiltration (data not published). This is confirmed by pathomorphological examination in previous studies of pigs of different ages and weights [25,27]. All regions in the present study showing increased signal intensities in T1-weighted images were traced back to inflammatory reactions due to the injection. As all injections (except of the immunological product) were given into the ham muscle, interactions with other local reactions can be excluded.

Vol_diff´s up to 3.36 cm^3^ were detected (Table 2) and linear measurements reached a maximum depth of 32.9 mm and a maximum length of 68.7 mm (Table 3). The maximum Vol_diff was detected at day 36 post injection and the maximum length at day 14 after booster injection. A comparison of linear and volume measurements is not possible. This can be explained by the image evaluation. Linear measurements were performed at the image with maximum reaction sizes. So linear measurements represent a single slice, whereas volume calculations show the distribution of the reactions in neighboring slices, which might be different. Additionally, it has to be kept in mind, that different sequences had to be used due to the animal´s growth. Nevertheless, a large local tissue reaction was detected. This was confirmed by the SPC of the immunologic product [15] and by a previous study [24]. In contrast to Bernau et al. [24], the maximum reaction size was evaluated at day 36 post injection instead of day 1. One reason for that could be a different injection type or size of animal.

The calculated penetration depth was measured in 14.3 ± 3.8 mm under the skin surface (Table 3), which fits with the prescribed penetration depth for this product. This penetration depth resulted in affected muscle tissue as shown in MR images (Figure 2). This is in contrast to the prescribed injection tissue. This product must be injected into subcutaneous tissue. Therefore, a safety vaccinator should be used, guaranteeing a subcutaneous injection [15]. Although the safety vaccinator and the prescribed needle length (19 mm) were used in the present study, muscle tissue was affected. This might have been be due to the pressure of the injection. Therefore, there is a need to evaluate the optimum needle length when using the safety vaccinator and using animals of different sizes. An incorrect needle length might result in large and long-lasting local reactions, distributions along the fascia or in a loss of efficacy. This is of major concern as food-producing animals are injected for prophylactic or curative reasons several times. 

Besides the kind of injection, long lasting tissue reactions were detected. These large reactions might have been due to the adjuvant. It is well known that the adjuvant influences the process of local reaction by producing an inflammation of variable size depending on the adjuvant used [32,33,34]. The adjuvant used in this immunologic product is DEAE Dextran, which is reported to be an effective adjuvant for improving the immunogenicity of vaccines [35,36,37]. It shows a dose related effect on neutralizing titer and a significant increase in serum antibody response [36,37,38]. This injection resulted in a long-lasting local reaction detectable using MRI until day 36 after the first injection (Table 2). It has to be kept in mind that the local reaction after the booster injection might have affected carcass quality due to long-lasting local tissue alterations. Neither Wittmann et al. [37] nor Einarsson [39] observed any tissue reactions in pigs after slaughter. To fully evaluate the impact of immunologic castration, the effect of the booster injection on carcass quality should be examined in further research with suitable methods able to identify the injection site exactly.

The detected local tissue reactions in this study are in contrast to previous studies, using palpation of the injection site to evaluate local tissue reactions [39,40,41]. McGlone et al. [42] did not find any reaction at the injection site although it was reported that cellular damage away from the injection site can be induced [43]. It was proven, that MRI can detect local tissue reactions [24,25,26,27], which was confirmed by pathomorphological examination [25,27] although in most cases no macroscopic tissue changes were detected on the skin surface. Therefore, to evaluate local tissue reactions, palpation of the injection site and macroscopic skin observations may not show their full extent. In pigs especially, evaluating the skin tissue can only give insights to fighting behavior, not into local tissue reactions. Imaging methods like MRI can be used as methods to give insight into the body and carcass and therefore present more information on animal health, welfare and carcass quality. The present study demonstrated these possibilities for MRI, offering objective examinations of local tissue reactions in large farm animals. Using imaging methods in large farm animals to detect local tissue reactions would result in better diagnostics and accurate tests, giving insights into living animals and finally providing more animal welfare.

Based on the present findings, it seems necessary to evaluate the effect of large local reactions on animal health and welfare. In human medicine, it is already known that the length and the extent of increased signal alterations in MR images correlate with the severity of injury [44]. This has to be proven regarding local tissue reactions in animals as well in order to not over-interpret the tissue damage and its impact. Therefore, for further studies, behavioural changes and performance parameters should be included to evaluate the influence of the local reaction on food intake, weight gain and pain expression. Some behavioural studies on the use of immunocastration in pigs exist but all focus on mounting or fighting behavior in immunologically castrated males compared to physically castrated males (e.g., [12,13,14,45,46]). McGlone et al. [42] examined behavior 1 h before and 1 h after the injection of Improvac^®^, which might be too short to see alterations adapted from the injection. Based on our own previous studies [25,27], it seems necessary to evaluate the behaviour post injection in more detail and with special attention to the days following injection. This should be combined with an MRI examination and carcass examinations to evaluate the local reaction to the fullest extent. This is of special importance because the immunologic product is not only used as an alternative for surgical castration, but also in female pigs [9,10,11,47,48,49,50,51,52] and other farm animals [5,6,7,8].

## 5. Conclusions

To sum up, immunological castration resulted in long-lasting local reactions detected by MRI. But, further studies are needed to evaluate the impact of local reactions on animal welfare and weight gain; additionally, the impact on carcass quality should be examined. According to human studies, it has to be discussed how long local reactions are supposed to be “acceptable”, which size of reaction can be supposed to be “endurable” based on the benefit the special injection has, and how animal welfare issues related to large local reactions can be measured.

## Figures and Tables

**Table 1:** MRI parameters for the coronar T1-weighted sequence, divided into the examination times.

MRI Parameter	Scan 1	Scan 2 & 3
Time to Repeat (ms)	814	814
Time to Echo (ms)	17	17
Field Of View (mm)	230	350
number of slices	22	22
slice thickness (mm)	4	5
distance factor	0.5	1.00
examination time	5 min 40 s	5 min 40 s

**Table 2:** Results of the paired *t*-Test (*p*-value) divided into the different scans (1, 2, 3), including number of animals (*n*) at each scan. 5 MR images were evaluated and included in the Vol_diff calculation.

Scan	Weight (kg)	Days Post Injection	Vol_diff & SEE (cm^3^)	*n*	*p*-Value
1	27.1 ± 5.1	1	1.27 ± 0.29	34	0.0001
2	51.7 ± 8.7	36	3.36 ± 0.72	34	<0.0001
3	91.6 ± 11.1	14 (booster injection)	1.56 ± 0.65	22	0.0225

**Table 3:** Results of the linear measurements and the location score.

Variable	Scan 1	Scan 2	Scan 3
	1 d p.i.	36 d p.i.	14 d p.i. (booster)
	*n* = 34	*n* = 34	*n* = 22
location score	1.9 ± 0.2	1.9 ± 0.3	1.7 ± 0.4
mean maximum depth of local reaction (mm)	28.6 ± 6.3	32.7 ± 9.3	32.9 ± 10.4
mean maximum length of local reaction (mm)	53.6 ± 12.7	60.0 ± 24.6	68.7 ± 18.6
penetration depth at first injection (mm)	14.3 ± 3.8		

## Data Availability

Data sharing not applicable.
